# Longitudinal assessment between lifestyle-related risk factors and a composite cardiovascular disease (CVD) risk index among adolescents in Malaysia

**DOI:** 10.1038/s41598-021-98127-0

**Published:** 2021-09-27

**Authors:** Nithiah Thangiah, Tin Tin Su, Karuthan Chinna, Muhammad Yazid Jalaludin, Mohd Nahar Azmi Mohamed, Hazreen Abdul Majid

**Affiliations:** 1 Centre for Population Health (CePH), Department of Social and Preventive Medicine, Faculty of Medicine, University of Malaya, Kuala Lumpur, Malaysia; 2 South East Asia Community Observatory (SEACO), Global Public Health Jeffery Cheah School of Medicine and Health Sciences, Monash University, Selangor, Malaysia; 3 School of Medicine, Faculty of Health and Medical Sciences, Taylor’s University, Selangor, Malaysia; 4 Department of Paediatrics, Faculty of Medicine, University of Malaya, Kuala Lumpur, Malaysia; 5 Sports Medicine Unit, Faculty of Medicine, University of Malaya, Kuala Lumpur, Malaysia; 6 Department of Nutrition, Faculty of Public Health, Universitas of Airlangga, Surabaya, Indonesia

**Keywords:** Risk factors, Cardiovascular diseases

## Abstract

The study aims to create a composite risk index of CVD among adolescents and examine the influence of demographic, socioeconomic and lifestyle-related risk factors on the composite risk index of biological CVD risk factors among adolescents in Malaysia. A Malaysian adolescent cohort of 1320 adolescents were assessed at 13, 15 and 17 years. Seven biological CVD risk factors with moderate correlation were identified, standardized and averaged to form a composite CVD risk index. Generalised estimating equation using longitudinal linear regression was used to examine the effects of changes in adolescent lifestyle-related risk factors on the composite CVD risk index over time. From the ages 13 to 17 years, physical fitness (β = − 0.001, 90% CI = − 0.003, 0.00002) and BMI (β = 0.051, 95% CI = 0.042, 0.060) were significant predictors of attaining high scores of CVD risk. Female (β = 0.118, 95% CI = 0.040, 0.197), Chinese (β = 0.122, 95% CI = 0.006, 0.239), Indians (β = − 0.114, 95% CI = − 0.216, − 0.012) and adolescents from rural schools (β = 0.066, 95% CI = − 0.005, 0.136) were also found to be considerably significant. A more robust and gender-specific intervention programme focusing on healthy lifestyle (including achieving ideal BMI and improving physical fitness) need to be implemented among school-going adolescents.

## Introduction

In Malaysia, cardiovascular disease (CVD) is the number one killer and accounts for 21.65% and 23.79% of all ten principal causes of deaths occurred in government and private hospitals, respectively in 2018^1^. The prevalence is on the rise as total deaths in 2006 reported at 15.4% jumped to 25.4% in 2010^2,3^. The most prominent risk factors of CVD in Malaysia are hypertension, hypercholesterolemia and obesity that have been increasing in prevalence at 30.0%, 38.1% and 50.1% respectively in 2019^4^. Among children below 18 years old in Malaysia, the national prevalence of obesity in 2011 was 6.1% and increased to 11.9% in 2015^5,6^. As such, the mortality rates from CVD and escalating prevalence of CVD risk factors in Malaysia are anticipated to rise and double the CVD burden in the coming decades.

Modifiable risk factors have an indirect relationship with the increased prevalence and death from CVD. The INTERHEART study conducted across 52 countries found that 90% of the risk of preliminary acute myocardial infarction in young people was attributed to nine modifiable cardiovascular risk factors including cholesterol, blood pressure, waist to hip ratio, diabetes, physical activity, intake of fruits and vegetables, smoking, alcohol and depression/stress^7^. In 2008, WHO reported that cardiovascular deaths attributed to physical inactivity and, overweight and obese were 6% and 5% of global deaths, respectively^8^. Additionally, in 2012, physical inactivity and unhealthy diet were identified as major risk factors of CVD^9^. These lifestyle risk factors are responsible for about 80% of coronary heart disease and cerebrovascular disease. In 2017, more than 50% of school-going adolescents in Malaysia spend at least 3 h sitting in a day and only 19.8% are physically active for at least 60 min in a day^10^. The effects of unhealthy diet and physical inactivity may manifest in individuals in the form of raised blood pressure, raised blood glucose, raised blood lipids, overweight and obesity can be classified as biological CVD risk factors^11^.

Since the formation of atherosclerotic lesions start from young, it is important to study the longitudinal determinants of these clustered biological CVD risk factors among adolescents. Although genetic-risk have known to cause obesity and other CVD risks^12^, lifestyle-changing factors should be identified early to have a more significant impact in reducing the risks of CVD later in life rather than the effort taken to educate older adults on preventive measures when it is perhaps a little too late^13^. Therefore, the objective of the study was to create a composite risk index for CVD among adolescents by averaging the standardized biological CVD risk factors and to examine the longitudinal relationship between lifestyle-related risk factors and the composite risk index for CVD among adolescents in Malaysia from the age of 13 to 17 years old.

## Methods

The STROBE statement for cohort was adhered in reporting this study.

### Participants

The Malaysian Health and Adolescents Longitudinal Research Team study (MyHeARTs) is one of the first initiatives among low and middle income countries to examine the trends of risk factors of NCD in an adolescent closed cohort study. The respondents were followed from the ages of 13, 15 and 17. A two-stage stratified cluster sampling design was used in this study. In the first stage, 15 schools (8 urban-based and 7 rural-based) were randomly selected from a complete list of secondary public schools located in the Federal Territory of Kuala Lumpur (FTKL) and the central and northern zone of Peninsular Malaysia. As the capital city of Malaysia, FTKL is an urban area and is chosen to well represent urban schools in Malaysia. The northern zone represents predominantly rural schools. In the second stage all 13-year-old students from the selected schools were invited to enrol in the study. More details about the study procedure and sampling have been reported by Hazreen et al. (2014) which reports findings from the data collected during the study baseline^14^. There were 1361 students who participated in the study at baseline. In the follow-ups there were 925 and 654 students in 2014 and 2016 respectively. In 2012, out of the 1361 students, 1,320 students had complete measurements for all the biological CVD risk factors considered in this study whereas in 2014 and 2016, it was 881 and 637 respectively. All procedures involving human subjects were approved by the Ethics Committee of University Malaya Medical Centre (Ref. No. 14-376-20486). All methods were performed in accordance with the relevant guidelines and regulations. All parents of participating children had signed and provided informed consent form.

### Measurements

#### Dependent variable

The outcome variable is a composite risk index for CVD representing the overall CVD risk score including seven biological CVD risk factor measurements that were standardised (transformed into z-score) and averaged^15^ for each survey year. Among the CVD risk factors considered were systolic blood pressure, diastolic blood pressure, body fat, total cholesterol: HDL ratio, HDL cholesterol, LDL cholesterol and triglyceride. Details of each measurement are described briefly as they have been presented elsewhere^14^. Medical doctors took the reading of systolic and diastolic blood pressure using a stethoscope and a mercurial sphygmomanometer (CK-101C, Spirit Medical Co., Taiwan). The percentage of body fat was measured using the Tanita portable Body Composition Analyzer SC-240 MA^16^. The students were asked to fast for at least 10 h prior to blood taking by a phlebotomist. The 15 millilitres of blood samples withdrawn were processed at the field laboratories to measure fasting lipids (Advia Chemistry, Siemens, Germany—triglyceride, total cholesterol, high density lipoprotein cholesterol and low-density lipoprotein cholesterol).

#### Independent variables

Self-administered student questionnaire and parental questionnaire was distributed to students and parents respectively to gather information on demographic and socioeconomic variables. The demographic variables include gender, ethnicity and locality whereas the socioeconomic variables include family income (less than RM1500, RM1501-RM3000, RM3001-RM5000, more than RM5001), parental highest education (low, medium, high) and father’s employment status (employed, unemployed). After controlling for these variables, the longitudinal analysis measured the influence of lifestyle-related risk factors including physical fitness and BMI on the outcome variable. All the lifestyle-related risk factors were treated as continuous variables for the purpose of analysis. The physical fitness test was assessed by trained sports physicians using the modified Harvard Step test protocol. The total duration of exercise in seconds and the peak pulse rate of each student upon completion of each minute on and off the step box was recorded using the Fingertip Pulse Oximeter (Baseline 12-1926 Fingertip Pulse Oximeter, Fabrication Enterprises Inc., USA). The physical fitness score was calculated by the total duration of exercise in seconds × 100 and divided by the sum of heart rates taken at 0, 1 and 2 min of rest. The final score was applied as a continuous variable in the statistical analysis. Theoretically, physical fitness is a more objective measurement that gauge’s endurance and agility to better reflect ones’ active physical status. Body mass index was calculated as weight in kilograms divided by the square of height in meters (kg/m^2^) and was applied as a continuous variable in the statistical analysis. Students’ height was measured using a stadiometer (Seca Portable 217, Seca, UK) and was recorded to the nearest 0.1 cm. Students’ weight was measured using a digital electronic weighing scale (Seca 813, Seca, UK) and was recorded to the nearest 0.1 kg. The dietary intake of the adolescents was measured using an open-ended 7-day diet recall questionnaire conducted by trained dietitians. The intake of fat and protein was expressed as a percentage of total daily intake of energy. The dietary intake needed to be included in the analysis as a confounder to ensure that the effect of other lifestyle-related risk factors on the average CVD risk score is controlled for macro- and micronutrients.

### Statistical analysis

Descriptive statistics for all demographic and socioeconomic variables were reported in the form of frequency and percentages. All biological CVD risk factors and lifestyle-related risk factors were presented in mean and standard deviations as they were within acceptable kurtosis and skewness range. Independent samples t-test were conducted to present significant mean differences between male and female adolescents. The sample size by each year was based on complete data available for the biological CVD risk factors. Based on Little’s MCAR test, missing data in the independent variables were not missing completely at random (MCAR), thus multiple imputation (MI) was done to. A total of 30 imputations and 100 iterations substituted all missing data. Upon imputation, a longitudinal linear regression using generalised estimating equations^17^ was used to determine factors associated with the overall CVD risk score among adolescents with repeated measurements in three durations from 13 through 17 years. The overall CVD risk score is a reasonable representation of cardiometabolic health regardless of the number of risk factors included^18^. The higher the overall CVD risk score, the higher the chances of being at risk of CVD. In lieu of the study objective, the main interest was to measure the effect of lifestyle-related risk factors on the agglomeration of combined outcome risk factors. Thus, a saturated model that considers the maximum number of all probable determinants was necessary. The time-dependent variables are time and lifestyle-related risk factors (BMI, PFS and dietary intake) whereas the time-independent variables are gender, ethnicity, locality and parental education. Parental employment and family income were also considered as time-independent for this study as only baseline information was available. The advantage of using GEE is that all available longitudinal data are used in one analysis producing regression coefficients (β) that indicates the relationship between lifestyle-related risk factors and the overall CVD risk score along the entire longitudinal period^19^. Besides that, GEE corrects for the fact that all repeated observations of the same subject are dependent on one another using a robust sandwich estimator. The correction for these within-subject correlations is done through the choice of an unstructured working correlation structure to allow for all possible correlations between repeated measurements^20^. Multicollinearity was tested for all lifestyle-related risk factors and upon removing carbohydrate, the variance inflation factors (VIF) for all variables were below 5 signalling no issues of multicollinearity. The level of statistical significance was set at a p-value of < 0.05. The IBM SPSS Statistics (version 22; SPSS Inc., Chicago, IL, USA) was used for all analyses.

## Results

Table 1 presents the demographic and socioeconomic characteristics of the adolescents and their parents only with complete data of the outcome variable for the three years of survey. The composition of female, Malay, urban and adolescents from Perak was comparatively higher in each year. Majority of students were from households with income less than RM 1500, parents with low to medium education and working fathers.

**Table 1:** Demographic and socioeconomic characteristics of adolescents by year.

Characteristics	13 years (n = 1320)	15 years (n = 881)	17 years (n = 637)
	N	N	N
**Gender**			
Male	509 (38.6)	303 (34.4)	183 (28.7)
Female	811 (61.4)	578 (65.6)	454 (71.3)
**Ethnicity**			
Malay	1077 (81.6)	692 (78.5)	491 (77.1)
Chinese	99 (7.5)	66 (7.5)	55 (8.6)
Indian	103 (7.8)	79 (9)	59 (9.3)
Others	41 (3.1)	44 (5)	32 (5)
**Locality**			
Urban	695 (52.7)	488 (55.4)	341 (53.5)
Rural	625 (47.3)	393 (44.6)	296 (46.5)
**States**			
Selangor	445 (33.7)	246 (27.9)	169 (26.5)
WPKL	282 (21.4)	176 (20)	127 (19.9)
Perak	593 (44.9)	459 (52.1)	341 (53.5)
**Family income**			
Less than RM 1500	546 (46)	405 (49.5)	318 (53.8)
RM 1501 to RM 3000	344 (29)	240 (29.3)	161 (27.2)
RM 3001 to RM 5000	136 (11.4)	86 (10.5)	64 (10.8)
More than RM 5001	135 (11.4)	68 (8.3)	36 (6.1)
**Parental highest education**			
Low	419 (38.3)	310 (41)	228 (41.9)
Medium	431 (39.5)	304 (40.3)	226 (41.5)
High	242 (22.2)	141 (18.7)	91 (16.8)
**Father employment**			
Employed	980 (92.3)	675 (90.2)	490 (93.0)
Unemployed	82 (7.7)	73 (9.8)	37 (7.0)

Table 2 displays the descriptive summary by gender for the selected CVD risk factors in the cohort of adolescents at the ages of 13, 15 and 17 years in 2012, 2014 and 2016, respectively. The average blood pressure was consistently and significantly higher among the males compared to females for all three years. On the other hand, blood lipid measurements were lower among males compared to the females although the ratio of total cholesterol to high density lipoprotein and triglyceride readings were not significantly different between genders in all years. In terms of body composition, the mean body fat percentage was significantly much higher among the female adolescents at all three time points. Physical fitness was significantly higher among the male adolescents at all three ages in this study. Body mass index was marginally higher among female adolescents although no significant difference was observed between gender at 13 years. Energy intake was significantly higher among the males throughout the study period. The percentage of macronutrient from total daily energy intake for protein and fat were within recommended values for both male and female adolescents in all three years.

**Table 2:** Descriptive summary for risk factors of adolescents by gender and year.

Risk factors	2012 (n = 1320)		2014 (n = 881)		2016 (n = 637)	
	Male (n = 510)	Female (n = 810)	Male (n = 303)	Female (n = 578)	Male (n = 182)	Female (n = 455)
	Mean ± SD	Mean ± SD	Mean ± SD	Mean ± SD	Mean ± SD	Mean ± SD
**Biological CVD risk factors**						
Systolic blood pressure (mmHg)	110.65 ± 10.95	108.53 ± 11.16	109.54 ± 12.72	104.05 ± 12.57	111.86 ± 11.60	107.08 ± 12.15
Diastolic blood pressure (mmHg)	68.88 ± 10.49	67.05 ± 10.26	66.99 ± 10.15	64.76 ± 9.82	68.62 ± 8.54	65.81 ± 9.16
High density lipoprotein cholesterol (mmol/L)	1.45 ± 0.31	1.49 ± 0.30	1.35 ± 0.30	1.47 ± 0.29	1.36 ± 0.26	1.54 ± 0.29
Total cholesterol:HDL ratio	3.22 ± 0.77	3.23 ± 0.70^§^	3.39 ± 0.90	3.33 ± 0.73^§^	3.18 ± 0.72	3.12 ± 0.71^§^
Low density lipoprotein cholesterol (mmol/L)	2.68 ± 0.72	2.77 ± 0.71	2.62 ± 0.73	2.89 ± 0.71	2.47 ± 0.75	2.74 ± 0.69
Triglycerides (mmol/L)	0.87 ± 0.38	0.92 ± 0.38	0.92 ± 0.45	0.87 ± 0.33^§^	0.86 ± 0.38	0.81 ± 0.33^§^
Body fat %	18.54 ± 14.41	25.72 ± 10.46	15.85 ± 11.13	29.42 ± 8.88	14.13 ± 8.82	28.66 ± 7.96
**Lifestyle-related risk factors**						
Physical fitness score	72.48 ± 10.69	62.66 ± 10.26	71.99 ± 13.06	51.60 ± 18.03	103.32 ± 18.80	73.79 ± 19.97
Body mass index (kg/m^2^)	19.67 ± 4.85	19.69 ± 4.40^§^	20.68 ± 4.93	21.37 ± 4.74	21.06 ± 4.91	21.89 ± 5.03
**Dietary intake**						
Energy (kcal)	1553.70 ± 420.78	1393.17 ± 402.30	2006.80 ± 637.76	1655.94 ± 534.04	2041.24 ± 565.94	1605.64 ± 460.23
Sodium (g)	2215.18 ± 787.06	1937.48 ± 703.62	2712.93 ± 1129.08	2428.54 ± 1246.09	2890.13 ± 1514.76	2745.70 ± 5498.32^§^
Cholesterol (g)	214.59 ± 105.77	180.78 ± 85.69	260.79 ± 128.74	213.23 ± 112.33	242.05 ± 126.77	192.82 ± 95.04
Protein as % energy	14.75 ± 2.35	14.82 ± 2.27^§^	14.93 ± 2.57	15.30 ± 2.26	13.72 ± 2.59	14.69 ± 2.56
Fat as % energy	29.85 ± 6.18	29.63 ± 4.58^§^	30.17 ± 4.73	31.93 ± 5.24	28.64 ± 6.04	31.46 ± 5.04

All measurements of biological CVD risk factors and lifestyle-related risk factors were significantly different between male and female adolescents except for values with ^§^ indicates not significant.

**Figure 1:**
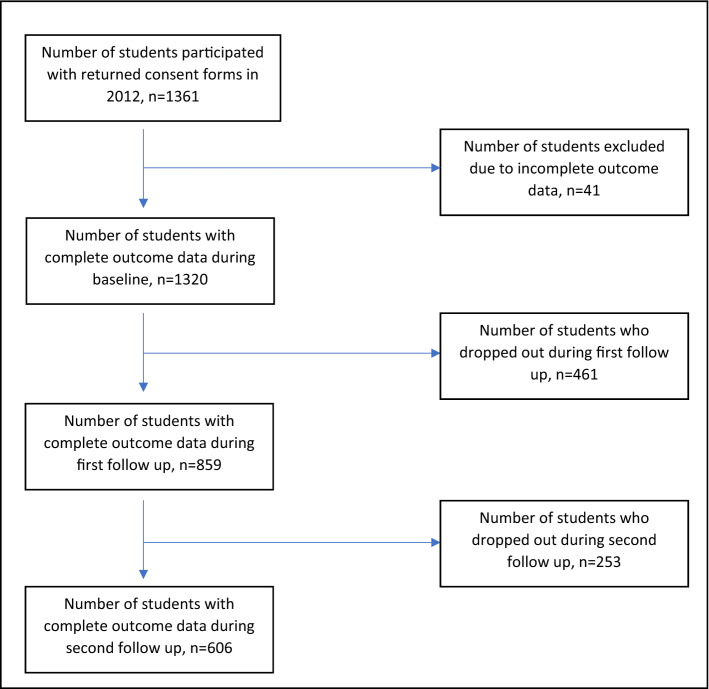
Number of students participated, dropped out and followed up from 2012 to 2016.

Figure 1 shows the sampling flow of the study to include the number of students who participated, were excluded, and dropped out from the single cohort of longitudinal analysis from the adolescents were 13 years old (year 2012) through 17 years old (year 2016). A total number of 606 students who participated from baseline until the second follow up and had complete data were included for the final analysis. The dropouts were mostly females, Malays and from urban schools. Independent samples t-test showed that the mean values of all the risk factors with respect to baseline lifestyle-related risk factors of each survey year did not differ between responders and dropouts.

Table 3 shows the results of the saturated model. Significant determinants on the overall CVD risk score for the ages between 13 to 17 years were the second follow up, time 3 (β = − 0.095, CI = − 0.154, − 0.035) and female (β = 0.118, CI = 0.040, 0.197). The results also showed that females were at higher risk compared to males. As compared to Malays, the average risk score among Indians (β = − 0.114, CI = − 0.216, − 0.012) and Chinese (β = 0.122, CI = 0.006, 0.239) were significantly lower by 0.114 units and higher by 0.122 units, respectively. Adolescents from rural schools (β = 0.066, CI = − 0.005, 0.136, p < 0.10) seem to have 0.066 units higher of overall CVD risk score compared to adolescents from urban schools. In terms of lifestyle-related risk factors, physical fitness score (β = − 0.001, CI = − 0.003, 0.0002, p < 0.10) and BMI (β = 0.051, CI = 0.042, 0.060) were also significant predictors of the overall CVD risk score, after controlling for the demographic, socioeconomic and dietary intake variables. Body mass index was the strongest predicting factor over time for these adolescents from 13 to 17 years.

**Table 3:** Results of saturated GEE model to assess the longitudinal association between composite CVD risk index and lifestyle-related risk factors among adolescents.

Risk indicator	13 to 17 years (n = 606)				
	β	SE	95% confidence interval		p-value
			Lower	Upper	
Intercept	−0.858	0.1718	−1.196	−0.521	< 0.001
**Time**					
T3	−0.095	0.0304	−0.154	−0.035	***0.002***
T2	−.043	0.0264	−0.094	0.009	0.106
T1	0^a^				
**Gender**					
Female	0.118	0.0401	0.040	0.197	***0.003***
Male	0^a^				
**Ethnicity**					
Others	0.094	0.0833	−0.069	0.258	0.258
Indian	−0.114	0.0521	−0.216	−0.012	***0.029***
Chinese	0.122	0.0596	0.006	0.239	***0.040***
Malay	0^a^				
**Locality**					
Rural	0.066	0.0360	−0.005	0.136	*0.068*
Urban	0^a^				
**Family income**					
More than RM 5001	0.071	0.0725	−0.071	0.213	0.330
RM 3001–RM 5000	0.061	0.0563	−0.049	0.172	0.276
RM 1501–RM 3000	0.0005	0.0444	−0.087	0.087	0.991
Less than RM 1500	0^a^				

Risk indicator	13 to 17 years (n = 606)				
	β	SE	95% confidence interval		p-value
			Lower	Upper	
**Parental highest education**					
High	−0.097	0.0619	−0.219	0.024	0.116
Medium	−0.022	0.0434	−0.107	0.063	0.618
Low	0^a^				
**Father employment**					
Employed	−0.086	0.0719	−0.227	0.055	0.230
Unemployed	0^a^				
**Dietary**					
Energy	−0.00006	0.00004	−0.0001	0.00002	0.168
Protein (as a percentage of energy)	−0.001	0.0055	−0.011	0.010	0.916
Fat (as a percentage of energy)	−0.0005	0.0026	−0.006	0.005	0.863
Physical fitness score	−0.001	0.0008	−0.003	0.0002	*0.087*
Body mass index	0.051	0.005	0.042	0.060	** < *0.001***

Estimates from sodium and cholesterol were extremely small and insignificant, hence are not shown.

Values in bold and highlighted are significant at 0.05 and values that are highlighted are significant at 0.10.

Quasi-likelihood under independence model criterion (QIC)=23,529.49.

Corrected quasi-likelihood under independence model criterion (QICC)=23,476.13.

Although the results of the regression model present neither of the socioeconomic status or dietary intake as significant determinants for the adolescent CVD risk score, the power of the study was deemed sufficient considering the attrition rates were at acceptable ranges. In addition, these variables were needed to be included as confounding factors.

## Discussion

The current study analyses the longitudinal association between overall CVD risk score and lifestyle-related risk factors controlled for demographic, socioeconomic factors and dietary intake among adolescents. The study duration is over 5 years with two follow ups. To our knowledge, this is the first longitudinal adolescent study to analyse the association between CVD risk score and lifestyle-related risk factors in Malaysia. In-depth reviews on the determinants of cardiometabolic health among Malaysian adolescents highlight the lack of longitudinal studies conducted in Malaysia. Most studies found the associations with diet and physical activity inconsistent and often small^21,22^.

The result from this study shows that compared to male, female adolescents pose a higher risk of CVD. The estimates for female adolescents demonstrated significant positive associations with overall CVD risk score increase by 0.118 units more than males from 13 to 17 years. The trend in body fat among female adolescents in this study found increases in body fat from 25% at 13 years to 29% at 15 years and 28% at 17 years. Increased fat mass over the years may induce more stress onto the female cardiovascular system. In contrast, male adolescents experienced a reduced body fat percentage from 18 to 14% from 13 to 17 years. Even the NHMS Adolescent Health Survey in 2017 reported more females to be overweight than males^10^ although actual body composition was not reported. Besides that, physical fitness score was found to be higher among male compared to female adolescents^10^. Clearly male adolescents lead a more active lifestyle than female adolescents hence making them to be fitter with lesser body fat than their female counterparts. In addition, the mean score of physical activity in this study was consistently in the lowest category (less than 2.33) among female adolescents from 13 to 17 years. This concurred with NHMS 2017 reports of significantly lower prevalence of physical activity among female (35.2%) compared to male (54.1%)^10^. In terms of sitting activities (at least 3 h of sitting activity in a usual day), the prevalence was higher among female at 52.3% whereas male at 47.9%^10^. Furthermore, national findings also reported a decline in adolescents being active at least 60 min in a day for five days consecutively from 22.7% in 2012 to 19.8% in 2017^10^. The lack of participation in outdoor activities due to cultural constraints and low quality physical education during school could perhaps be the reason for the sedentary behaviours^23^. A few other studies from the same cohort of MyHeARTs reverberated the reason why female adolescents are at increased risk of developing CVD than male adolescents. Lack of vitamin D impairs bone growth and increases the risk of developing CVD and Quah et al. (2018) found female adolescents more likely to have vitamin D deficiency^24^. Another possible explanation for higher risk of CVD among females compared to males is explained by lower values of hand grip strength among females from the same study cohort where poor muscle strength was reported to cause CVD^25^. The MyHeARTs cohort had also reported significant increases in the trend of anaemia prevalence among females where anaemia is also a potential risk factor for CVD^26^. In a nutshell, the findings from past studies of the same cohort lead to various possibilities to explain the reason behind the higher chances of attaining CVD among female adolescents in Malaysia compared to males.

A further analysis showed that ethnicity played an important role in determining the overall risk of CVD among adolescents. This study found that compared to Malays, Chinese adolescents were more likely to develop CVD over time. A possible explanation by Soh et al. (2011) found high prevalence of internet use leading to long sedentary hours, physical inactivity and weight gain among Chinese adolescents in Malaysia^27^. Evidence can also be linked to national reports of adolescents from Chinese ethnic having the lowest prevalence of physical activity and highest prevalence of obesity in 2015 among children below 18 years in Malaysia^10^. On the other hand, Indian adolescents were less likely to be at risk of CVD compared to Malays. Evidence from reports in the NHMS Adolescent Nutrition Survey in 2017 reported Indians to be the most active^28^. However, further research is needed to examine the possibility of genetic factors or differences in food intake that may contribute to the likelihoods based on ethnicity. Adolescents from rural schools were found to have higher chances of being at risk of CVD. Since a substantial part of students’ daily food consumption is from school canteens and vendors from outside the school area^28^, stringent dietary guidelines should be imposed to ensure healthy food is available for school-going adolescents. From this study, none of the parental socioeconomic status were found to contribute to CVD risk although these were important to be included as confounding variables to examine the influence of lifestyle-related risk factors on CVD. Nevertheless, insignificance could be attributed to small sample size or low levels of parental supervision as was reported in the NHMS Adolescent Health Survey in 2017 to be only at 13.2%^10^.

Besides female adolescents being at high risk of CVD, the determinant on physical fitness score was found to be a consistent predictor for the overall CVD risk score over the 5 year period from 13 to 17 years. Adolescents with increasing physical fitness score reduced their risk of attaining CVD over time. A higher physical fitness score can be obtained through frequent exercising that benefits the physiological health. With regular exercise, the heart strengthens and can pump with a higher force that regulates an enhanced blood flow. This allows the heart to beat less frequently but with increased blood flow throughout the system. Although physical fitness score was a consistent determinant, the estimates were rather small. The NHMS Adolescent Health Survey in 2017 reported only about 1/5 (19.8%) adolescents were physically active for at least 60 min in a day for five days in the past week compared to 22.7% in 2012^29^. Similarly, a study on Early Child Care and Youth Development found physical activity decreasing significantly among children from 9 to 15 years^30^.

The Malaysian Dietary Guidelines and the WHO’s guidelines on physical activity for children and adolescents aged 5–17 years^31,32^ recommends that daily activity of moderate or vigorous intensity should be at least 60 min. The Muscatine Study and the Cardiovascular Risk in Young Finns Study proved that longitudinal assessments of physically active individuals presented optimum cardiovascular risk profiles^33,34^. It is recommended that adolescents should avoid being sedentary for more than an hour at any time. Unfortunately, more than half of the adolescents from the MyHeARTs study were found to be physically inactive.

Among all variables, the most intriguing was BMI that remained persistently as the most significant and strongest predicting factor in the cohort studied and this finding echoes many other Asian studies^35–38^. Although BMI may have direct associations with the outcome variable that includes body fat among other risk factors such as blood pressure and cholesterol, but it is important to acknowledge the multiplicative effect of BMI on the clustered CVD risk factors as an agglomerated outcome. As CVD occurs due to interactions between multiple risk factors, the best method of determining its’ risk is by including the maximum probable predictors to prevent missing out on important variables. In fact, a recent study using data from the NHMS 2006, 2011 and 2015 reported significant associations between BMI and CVD risk levels especially in younger age groups^36^. Worse yet, among countries in Southeast Asia, Malaysian children and adolescents aged 5–19 years ranked second highest in ASEAN with 12.7% being obese according to the Global Health Observatory Data Repository, World Health Organisation. The trend of increasing obese levels, particularly among adolescents in Malaysia is alarming with elevated CVD risk. Much needs to be done to circumvent these adolescents from progressing into high risk during adulthood. As such, all lifestyle-related risk factors of CVD are inter-related and should be regarded simultaneously to avoid the risk of attaining CVD.

Policy recommendations should be implemented at all levels to ensure positive implications. The adolescent cohort form the best target group for intervention programmes related to CVD risks as many lifestyle behaviours are established during this period. The findings from this study highlight the importance of programme implementations especially at the school and government levels to have significant and reduced impacts of CVD. The school management can play an enormous part to address problems of inactivity and identification of weight gain especially in female adolescents by providing periodic health card reports through scheduled monitoring. Adolescents who do not adhere or meet acceptable standards should be closely monitored or referred to counselling and healthcare providers. The government on the other hand, should implement assertive strategies by strengthening and revisiting on-going Health Plan of Actions through nutritional and physical health interventions in adolescents^39^.

### Strengths & limitations

This study is one of the first to investigate the longitudinal clustering of CVD risk factors in a large cohort of adolescents in Asia. The longitudinal design of this study allows to establish causal relationships in explaining the determinants of CVD risk. Besides that, clustering of biological CVD risk factors to form a composite risk index is a good indicator to combine multiple measurements of an adolescents’ CVD risk. Evidence show that the combined risk factors have stronger associations with lifestyle-related risk factors^40^. Some limitations in the study assumes that all the biological CVD risk factors included are equally valued. Since there is unclear evidence on how risk factors, especially in children should be weighed as CVD is usually manifested only in adulthood, standardizing the risk factors was deemed most appropriate to overcome this problem^41^. Since only three states within Peninsular were chosen, this may limit the generalisability of the findings.

## Conclusion

Cardiovascular diseases has shown to develop from young thus lifestyle modification during adolescence is pertinent. The transitional time during adolescence is ideal for developing good practice of lifestyle habits and behaviours. The findings from this study suggest that interventions to reduce CVD risk should focus on achieving healthy weight while increasing physical activity with focus on gender-specific programmes. Healthy lifestyle interventions play a role in preventing the development of CVD.

## Data Availability

The dataset analysed for the current study is not publicly available due to participant’s confidentiality and privacy. Requests to access the datasets should be directed to the corresponding author.
